# A YOLOv8-Based Real-Time Road Congestion Decision-Making Approach Fused with Channel–Spatial Attention and Dynamic Weighted Loss

**DOI:** 10.3390/s26134299

**Published:** 2026-07-06

**Authors:** Wei Huang, Heyang Xu, Hao Bai, Le Yu

**Affiliations:** 1 Sichuan Expressway Construction and Development Group Co., Ltd., Chengdu 610041, China; quan28186@gmail.com (W.H.); sdigbh@126.com (H.B.); 2 School of Information Engineering, Yancheng Institute of Technology, Yancheng 224051, China; yule130@126.com

**Keywords:** traffic congestion decision making, UAV remote sensing, vehicle detection, YOLOv8, attention mechanism

## Abstract

Conventional object detection models suffer from significant performance degradation in dense urban traffic scenarios. To address these critical limitations and enable accurate real-time road congestion decision making, this study proposes an optimized YOLOv8-based detection paradigm that decouples multi-scale feature enhancement from dynamic focused bounding box regression. Specifically, a multi-scale feature enhancement (MFE) module is designed to extract high-resolution shallow features directly from the P2 layer of the YOLOv8 backbone. Then, a convolutional block attention module (CBAM) is embedded into the feature fusion neck to adaptively filter complex urban background noise and recalibrate channel–spatial feature responses for vehicle target saliency. Furthermore, the standard CIoU loss is replaced with the Wise-IoU (WIoU) dynamic focusing loss function, which suppresses gradient interference from low-quality, occluded samples and stabilizes bounding box regression for dense vehicle targets. The high-precision vehicle detection outputs are fed into a quantitative congestion index (CI) model, which fuses vehicle density and average speed to realize real-time congestion-level classification. Extensive experiments on the public UAVDT benchmark dataset demonstrate that the proposed model achieves an mAP@0.5 of 83.1% (3.8 percentage points higher than the YOLOv8 baseline), an mAP_S (small target) of 23.2% (a 4.3 percentage point improvement), and a real-time congestion decision accuracy of 83.8%. Ablation studies verify the independent and synergistic effectiveness of the MFE, CBAM, and WIoU modules, with the MFE module making the greatest contribution to small-target detection performance (+1.7% mAP@0.5). The proposed model maintains a real-time inference speed of 86 FPS (frames per second) on an NVIDIA RTX 3090 GPU, far exceeding the 30 FPS threshold for real-time traffic monitoring.

## 1. Introduction

Rapid urbanization and the exponential growth of motor vehicle ownership have made traffic congestion a global municipal infrastructure bottleneck, leading to increased travel time, energy consumption, and air pollution [1,2]. According to the 2024 Global Urban Traffic Congestion Report, the average peak-hour traffic congestion index of megacities worldwide has reached 0.65, with some densely populated urban areas exceeding 0.8 [3]. Traditional ground-based traffic monitoring methods (e.g., loop detectors, microwave sensors) suffer from limited coverage, high maintenance costs, and poor adaptability to complex road networks [4]. Unmanned aerial vehicles (UAVs) and fixed high-altitude surveillance platforms have emerged as core tools for intelligent traffic management due to their advantages of high mobility, wide field of view, low deployment cost, and real-time data acquisition [5]. Extracting accurate vehicle target information (position, count, speed) from UAV high-altitude imagery is the foundational prerequisite for evaluating traffic states and realizing automated congestion decision making [6,7,8].

Object detection is the core technology for UAV-based traffic information extraction, and single-stage detection models represented by the YOLO (you only look once) series have become the mainstream choice for real-time traffic monitoring due to their balance of accuracy and inference speed [9]. YOLOv8, the latest iteration of the YOLO series, adopts an anchor-free architecture, advanced C2f bottleneck modules, and decoupled classification-regression heads, achieving state-of-the-art performance on general object detection benchmarks (e.g., MS COCO) [10,11]. However, direct deployment of the YOLOv8 baseline for UAV high-altitude traffic surveillance tasks exposes three critical structural deficiencies. The first is feature dilution of small targets. Distant vehicles in UAV imagery occupy only a few pixels, and successive downsampling in the backbone network discards high-frequency spatial details required for small-target classification [12]. The second is complex background interference. Urban road scenes contain abundant noise (building shadows, lane markings, pedestrians, green belts), and the lack of explicit attention mechanisms in YOLOv8 leads to high false positive rates in vehicle detection [13]. The third is bounding box regression instability. Dense vehicle flow and severe occlusion generate a large number of low-quality samples, and the standard CIoU loss treats all samples homogeneously, leading to gradient descent deviation and poor regression accuracy [14]. These deficiencies result in low vehicle detection accuracy, especially for small distant targets, and further lead to lagging and inaccurate road congestion decision making [15].

Road congestion decision making is the core link connecting vehicle detection and ITS application, and its key is to design a scientific and effective congestion index (CI) [16]. Traditional congestion index models are mostly based on single indicators such as vehicle speed or density [17]. For example, Zhu et al. [18] propose a deep learning-based congestion detection model for urban intersections which used vehicle density as the only congestion indicator and achieved a decision accuracy of 79.2%. Mudaw et al. [19] use floating car data to predict traffic speed and identify congestion levels, with a decision accuracy of 80.5%. However, single-indicator models are easily affected by road conditions and vehicle types, and the decision results are not robust. Multi-indicator fusion models have become the mainstream development trend. Arthur et al. [20] propose a congestion index model fusing vehicle density, speed, and lane occupancy, achieving a decision accuracy of 82.1% on the Seoul traffic dataset. He [21] reviews short-term traffic flow prediction methods and points out that multi-indicator fusion can significantly improve the accuracy of congestion decision making. However, most existing multi-indicator models are designed for ground-based traffic monitoring data and do not fully consider the characteristics of UAV high-altitude monitoring data (e.g., high vehicle count accuracy, relatively low speed estimation accuracy). The weight assignment of indicators lacks empirical verification based on UAV data, and the real-time performance of the model needs to be further improved.

Based on the above research status, the main research gaps in the field of UAV-based real-time road congestion decision making are summarized as follows. First, most existing improved YOLOv8 models only focus on a single optimization direction and lack a comprehensive solution for the three core problems of feature dilution, background interference, and regression instability in UAV high-altitude vehicle detection. Second, traditional FPN/PAN-based fusion methods involve low participation of shallow high-resolution features, which cannot effectively solve the extreme feature dilution problem of small distant vehicles in UAV imagery. Third, the standard CIoU loss cannot handle low-quality, occluded samples in dense vehicle flow, and the adaptability of dynamic weighted loss functions (e.g., WIoU) to UAV traffic detection needs to be further verified. Fourth, most existing congestion index models do not consider the characteristics of UAV monitoring data in indicator weight assignment, and the real-time performance and robustness of the model need to be improved. Finally, most existing models are only verified on a single dataset, and the comparison with the latest state-of-the-art models is lacking, making it impossible to fully prove the effectiveness and generalization ability of the model.

To address the above problems, this study enhances the YOLOv8 model for the specific characteristics of UAV high-altitude traffic detection and designs a real-time congestion decision-making framework based on high-precision vehicle detection. The proposed multi-scale feature enhancement and attention mechanism optimization strategies enrich the research on YOLOv8 improvement for small-target detection in remote sensing imagery. The dynamic weighted loss function effectively solves the bounding box regression problem of low-quality samples in dense traffic scenarios, providing a new solution for object detection in complex scenes. The proposed congestion decision-making model, based on vehicle density and speed fusion, can be directly applied to UAV traffic surveillance systems, providing real-time, accurate traffic state information for ITS and supporting the intelligent upgrade of urban traffic management.

This study develops an enhanced YOLOv8-based real-time road congestion decision-making framework for UAV high-altitude traffic surveillance. Rather than claiming a new general detection paradigm, the contribution is positioned as a problem-oriented framework that integrates a lightweight P2-driven feature-fusion modification, attention-guided feature recalibration, dynamic bounding box regression, and a UAV-adapted congestion index. The specific contributions are as follows.

To clarify the scientific scope of the work, this manuscript does not claim CBAM, WIoU, ByteTrack, or weighted CI modeling as individual new algorithms. The contribution lies in the UAV-traffic-specific arrangement and validation of these components, especially the lightweight P2-P3-P4 MFE feature-flow path and the experimentally verified link between improved detection quality and congestion decision accuracy.

First, a problem-driven enhancement framework for UAV traffic detection is constructed. The MFE, CBAM, and WIoU components are arranged to correspond to three empirically observed degradations in UAVDT data: small-object feature dilution, complex-scene false positives, and regression instability for occluded or low-quality boxes. The framework improves detection accuracy while preserving real-time inference.

Second, a UAV-specific multi-scale feature enhancement (MFE) module is introduced. Unlike generic FPN/PAN/BiFPN aggregation, MFE explicitly injects shallow P2 detail features into the neck through a short P2-P3-P4 path and uses lightweight 1 × 1 dimensional alignment, thereby emphasizing small vehicle geometry while limiting extra parameters.

Third, a real-time congestion decision model adapted to UAV monitoring data is validated. The CI model fuses density (alpha = 0.6) and speed (beta = 0.4), and this paper adds confidence intervals, sensitivity tests, speed-estimation validation, and comparisons with existing traffic-state estimation baselines to clarify the contribution of the decision-making component.

The rest of this paper is organized as follows. Section 2 reviews related work on UAV-based vehicle detection, feature fusion, attention mechanisms, loss functions, and traffic-state estimation. Section 3 explains the YOLOv8 baseline only to the extent needed for the present design and analyzes the data-driven limitations observed in UAV traffic scenes. Section 4 presents the proposed enhancement framework, including MFE, CBAM, WIoU, ByteTrack-based speed estimation, and the CI decision model. Section 5 reports comparative experiments, ablations, diagnostic analyses, graphical interpretation, and limitations. Finally, Section 6 concludes the paper and clarifies the specific contributions and future work.

## 2. Related Work

In recent years, many efforts have been focused on the topic of the remote sensing-based vehicle detection of intelligent transportation. Researchers have conducted extensive research on improving deep learning detection models to adapt to the characteristics of UAV high-altitude imagery (small targets, dense clustering, complex backgrounds). For example, Lin et al. [22] proposed the UAVDT benchmark dataset, the first large-scale UAV aerial traffic object detection dataset, which laid a foundation for the evaluation of UAV-based vehicle detection models. Mudawi et al. [19] proposed a lightweight context-aware DCS-YOLOv8 model for small-target detection in UAV remote sensing imagery, which improved the mAP_S by 3.2 percentage points by adding a context enhancement module. Xu et al. [23] integrated a hybrid spatial feature pyramid network (HS-FPN) and CBAM into YOLO11, achieving an mAP@0.5 of 81.7% on the UAVDT dataset for infrared vehicle detection. Yang et al. [24] proposed the MSConv-YOLO model based on YOLOv8, which improved small-target detection performance by designing a multi-scale convolution module to enhance shallow feature expression. However, most existing models only focus on a single optimization direction (e.g., feature fusion or attention mechanism) and lack an integrated enhancement framework for the three core problems of UAV traffic vehicle detection (feature dilution, background interference, regression instability). In addition, the detection results of most models are not directly linked to congestion decision making, and the practical application value in ITS is limited.

Multi-scale feature fusion is a key technology to solve the scale variation problem of object detection targets. The classic feature pyramid network (FPN) adopts a top-down fusion strategy, propagating deep semantic features to shallow layers via upsampling to enhance small-target detection performance [25]. The path aggregation network (PAN) added a bottom-up fusion path on the basis of FPN, realizing bidirectional feature propagation and improving the fusion effect of shallow detail features and deep semantic features [26]. Tan et al. [27] proposed the BiFPN network, which optimized the feature fusion path by removing redundant nodes and adding cross-scale residual connections, further improving the multi-scale feature fusion efficiency. Wu et al. [28] proposed a multi-scale feature fusion method for traffic scene small-target detection, which fused shallow high-resolution features with deep semantic features via cross-layer concatenation, achieving an mAP_S of 20.5% on the UA-DETRAC dataset. However, traditional FPN/PAN-based fusion methods are designed for general object detection and do not fully consider the extreme feature dilution of small distant vehicles in UAV imagery. The participation of shallow high-resolution features is low, and the problem of small-target detail loss is still prominent.

Attention mechanisms can adaptively recalibrate feature responses, highlight target regions, and suppress background noise and have been widely applied in object detection, image classification, and semantic segmentation [29]. The squeeze and excitation (SE) [30] module is the first channel attention mechanism, which adaptively adjusts the weight of each feature channel by modeling the interdependence between channels. The convolutional block attention module (CBAM) [31] extended the SE module to the spatial dimension, designing a dual attention mechanism of channel and spatial attention which achieved better performance than the single SE module in various computer vision tasks. Woo et al. [31] verified that the CBAM module can be easily embedded into various convolutional neural networks (CNNs) with almost no additional computational cost. Qiang et al. [32] applied the CBAM module to YOLOv8s for vehicle detection, reducing the false positive rate by 1.8% by suppressing background interference. Kang et al. [33] proposed the SE-CBAM-YOLOv7 model for small aircraft target detection, which improved the detection accuracy by 2.9% by fusing SE and CBAM attention mechanisms. However, the application of CBAM in UAV traffic vehicle detection is still in the exploratory stage, and there is a lack of research on the adaptive adjustment of attention mechanism parameters for the specific characteristics of UAV high-altitude imagery.

Bounding box regression loss functions directly determine the localization accuracy of object detection models. The traditional IoU loss [34] only considers the overlapping area of the predicted and ground-truth bounding boxes and cannot handle the non-overlapping case. To solve this problem, GIoU [35], DIoU and CIoU [36] were successively proposed, which added the distance, aspect ratio, and other geometric information between bounding boxes to the loss function, improving the regression accuracy. Tong et al. [37] proposed the Wise-IoU (WIoU) loss function with a dynamic focusing mechanism, which adaptively adjusts the loss weight according to the outlier degree of the samples, suppresses gradient interference from low-quality samples, and stabilizes the training process. Sun et al. [38] proposed the InterpIoU loss function based on interpolation optimization, which improved the regression accuracy of small bounding boxes by interpolating the overlapping area of the bounding boxes. Jha et al. [39] applied the WIoU loss function to infrared small-target detection, reducing the regression error by 4.1% compared with the CIoU loss. However, the application of dynamic weighted loss functions in UAV traffic vehicle detection is limited, and there is a lack of research on the adaptability of WIoU to dense, occluded vehicle targets in UAV imagery.

## 3. YOLOv8 Object Detection Algorithm

### 3.1. Basic Framework of YOLOv8

YOLOv8 is selected as the primary baseline because it provides a stable, open-source, anchor-free, real-time detector with a mature training pipeline and a favorable accuracy–speed trade-off for UAV edge deployment. Although newer YOLO variants and remote-sensing-specific detectors have recently appeared, many either lack unified public implementations, require heavier transformer or slicing pipelines, or do not provide an end-to-end congestion–decision interface. Using YOLOv8s therefore allows a fair assessment of whether lightweight feature-fusion, attention, and loss-function changes can improve UAV traffic detection without sacrificing the real-time constraint. Recent remote-sensing and traffic-video studies further show that YOLO-family detectors remain widely used baselines for intelligent transportation and vehicle tracking applications [40,41].

To rebalance the manuscript, this subsection now briefly summarizes only the YOLOv8 components that are directly affected by the proposed design; standard details of YOLOv8, CBAM, and WIoU are intentionally kept concise in later sections.

**Figure 1:**
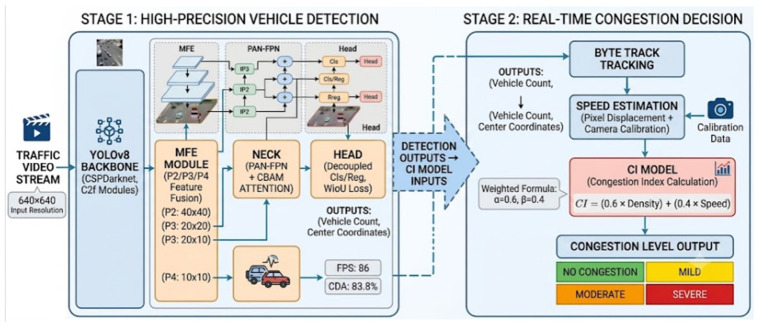
Overall framework of the enhanced YOLOv8-based traffic congestion decision system.

The YOLOv8 framework consists of three core parts, i.e., the backbone, neck, and head, as shown in Figure 1.

Backbone: The backbone network is based on the CSPDarkNet architecture and replaces the C3 module in YOLOv5 with the C2f module, which adds more residual connections and improves the feature extraction ability while maintaining the same computational complexity. The backbone network extracts multi-scale feature maps from the input image, which are denoted as P1 (80 × 80), P2 (40 × 40), P3 (20 × 20), P4 (10 × 10), and P5 (5 × 5) according to the resolution, from high to low. Shallow feature maps (P1–P2) contain rich spatial detail information (e.g., vehicle contour, wheel pixels), while deep feature maps (P3–P5) contain high-level semantic information (e.g., vehicle type, spatial position).

Neck: The neck network adopts a PAN–FPN hybrid structure, which realizes multi-scale feature fusion via top-down and bottom-up paths. The top-down path propagates deep semantic features to shallow layers via upsampling, and the bottom-up path propagates shallow detail features to deep layers via downsampling, fusing the advantages of both to improve the detection performance of multi-scale targets.

Head: The head network abandons the traditional anchor-based mechanism and adopts an anchor-free detection head, which directly predicts the center coordinates, width, and height of the bounding box, reducing the number of hyper-parameters and improving the generalization ability of the model. The head network is designed with a decoupled structure, which separates the classification and regression branches, and uses the BCE (binary cross-entropy) loss for classification and the CIoU loss + DFL (distribution focal loss) for bounding box regression, improving the training efficiency and detection accuracy.

The input resolution of the YOLOv8 model is set to 640 × 640 by default, which is suitable for UAV high-altitude imagery with medium and high resolution. The model adopts the Mosaic data augmentation method in the training stage, which splices four random images into one, enriching the training sample diversity and improving the model’s generalization ability. In the inference stage, the model adopts the NMS (non-maximum suppression) algorithm to remove redundant bounding boxes and output the final detection results.

### 3.2. Limitations of Baseline YOLOv8 in UAV High-Altitude Traffic Detection

**Figure 2:**
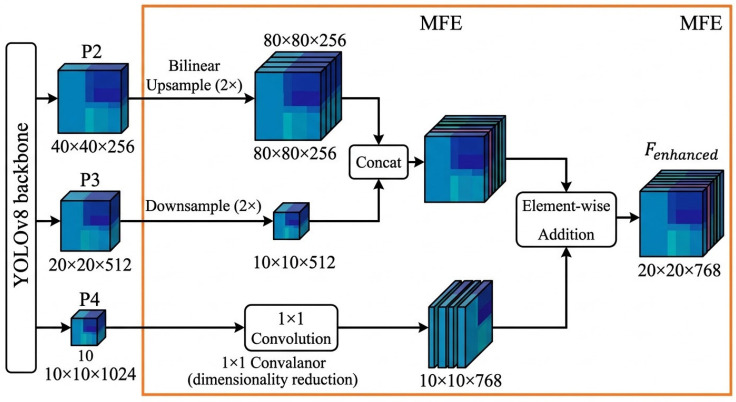
Structure diagram of the multi-scale feature enhancement module.

Although the YOLOv8 baseline achieves state-of-the-art performance on general object detection benchmarks (e.g., MS COCO), it exhibits significant structural deficiencies when deployed on UAV high-altitude traffic detection tasks, mainly reflected in three aspects: information dilution, background interference, and regression sensitivity, as shown in Figure 2.

#### 3.2.1. Information Dilution of Small Distant Vehicles

UAV high-altitude surveillance is characterized by a large field of view and a long detection distance, and distant vehicles in the imagery occupy only 5–20 pixels, which comprise typical small targets [12]. The YOLOv8 backbone network performs successive downsampling (total downsampling multiple of 32) on the input image, and the shallow high-resolution feature maps (P1–P2) containing small-target detail information are continuously compressed in the downsampling process. The traditional PAN–FPN fusion path in the neck network exhibits low participation of shallow P2 features, and most of the fused features are from deep P3–P5 feature maps with rich semantic information but lacking in detailed information. This leads to the severe feature dilution of small distant vehicles, and the model cannot effectively extract the geometric features (e.g., contour, texture) required for small-target classification, resulting in a high missed detection rate of small vehicles. Experimental results show that the mAP_S of the YOLOv8 baseline on the UAVDT dataset is only 18.9%, which is the main bottleneck restricting the overall detection performance.

#### 3.2.2. Complex Urban Background Interference

Urban road scenes captured by UAVs contain abundant complex background information, including building shadows, lane markings, green belts, pedestrians, non-motor vehicles, and other elements [23]. Some background elements (e.g., white lane markings, rectangular building shadows) express high pixel similarity with small vehicle targets, and the YOLOv8 baseline lacks explicit attention mechanisms in the feature fusion process. The model cannot effectively distinguish vehicle targets from background noise, and the receptive field is easily dominated by background regions, leading to a high false positive rate in vehicle detection. In addition, the dynamic changes in UAV flight attitude (e.g., pitch, roll) and lighting conditions (e.g., sunlight reflection, nighttime lighting) further increase the complexity of the background, making the model’s detection results unstable. Experimental results show that the false positive rate of the YOLOv8 baseline on the UAVDT dataset is 7.2%, which significantly affects the accuracy of vehicle count and speed estimation.

#### 3.2.3. Bounding Box Regression Sensitivity to Low-Quality Samples

Dense vehicle flow and mutual occlusion are common in urban road congestion scenes, and they generate low-quality training samples such as severely occluded vehicles, blurred distant vehicles, truncated vehicles at image boundaries, and boxes with uncertain annotation edges. In bounding-box regression, these samples are not merely difficult examples; they often produce noisy gradients because their center points, widths, heights, and aspect ratios deviate strongly from well-visible vehicles. When the standard CIoU loss assigns similar importance to all samples, the noisy gradients from such boxes can dominate mini-batch updates and reduce localization accuracy for small vehicles.

This effect is amplified in UAV imagery because many vehicles occupy only 5–20 pixels. A one- or two-pixel localization error can substantially change the IoU for a small vehicle, whereas the same absolute error has a much smaller impact on a large vehicle. Consequently, homogeneous CIoU weighting may over-penalize uncertain boxes and under-emphasize reliable boxes. The purpose of introducing WIoU is therefore not to discard difficult vehicles, but to dynamically reduce the harmful gradient contribution of extreme outliers while maintaining learning pressure on normal and moderately difficult samples.

### 3.3. Motivation for YOLOv8 Optimization

Aiming at the three empirically observed limitations of the YOLOv8 baseline in UAV high-altitude traffic detection, this study designs an integrated enhancement framework. The objective is to improve the detection of small and occluded vehicles while preserving real-time inference speed and to supply more reliable vehicle count and speed estimates for the downstream congestion decision model.

First, for information dilution, this paper designs a multi-scale feature enhancement (MFE) module to increase the participation of shallow high-resolution P2 features in multi-scale feature fusion. The MFE module directly extracts P2 features for upsampling and cross-layer fusion with P3/P4 features, effectively preserving the detail information of small distant vehicles and mitigating feature dilution during downsampling.

Second, for background interference, this paper embeds the CBAM attention mechanism into the YOLOv8 neck network to realize adaptive channel–spatial feature recalibration. The CBAM module highlights vehicle target regions and suppresses complex background noise, reducing the false positive rate of detection and improving the stability of the model.

Third, for regression sensitivity, this paper replaces the standard CIoU loss with the WIoU dynamic focusing loss function to realize the adaptive weight adjustment of samples. The WIoU module suppresses the gradient interference of low-quality samples and increases the loss weight of high-quality samples, stabilizing the bounding box regression process and improving the localization accuracy of small and occluded vehicles.

The three optimization modules are organically integrated into the YOLOv8 framework, and the synergistic effect between the modules is fully utilized to realize the comprehensive improvement of the model’s detection performance. The design of all modules follows the principle of lightweight design, and the additional parameters and computational cost are minimized to ensure the real-time inference speed of the model, which is suitable for UAV traffic surveillance tasks with limited computing resources.

## 4. Real-Time Road Congestion Decision Making Based on the Enhanced YOLOv8 Framework

This section details the proposed methodology for real-time road congestion decision making based on the enhanced YOLOv8 framework, including four core parts: the Congestion Index (CI) model, the multi-scale feature enhancement (MFE) module, convolutional block attention module (CBAM) integration, and WIoU dynamic focusing loss optimization. The pseudocode of the proposed UAV-based vehicle detection and congestion decision process is shown in Algorithm 1 and the overall framework is shown in Figure 1 and consists of vehicle detection followed by congestion decision making.
**Algorithm 1.** Pseudocode of the proposed UAV-based vehicle detection and congestion decision framework.InputUAV video frames I_t, camera calibration parameters, trained YOLOv8s–MFE–CBAM–WIoU weights, Nmax, Vfree, alpha = 0.6, beta = 0.4.1Resize each frame to 640 × 640 and apply the enhanced YOLOv8 detector to obtain vehicle boxes, confidence scores, and classes.2Use MFE-enhanced features and CBAM-recalibrated neck features to reduce missed small vehicles and false positives.3During training, compute WIoU + DFL for box regression; during inference, decode boxes and apply NMS with the configured threshold.4Pass detected vehicle boxes to ByteTrack, and associate vehicle identities across adjacent frames.5Convert center-point displacement to real speed using calibration factor k, and apply a three-frame moving-average filter.6Compute density D = clip(N/Nmax, 0, 1), speed reduction S = clip(1 − V/Vfree, 0, 1), and CI = alpha × D + beta × S.7Map CI to four traffic states: no congestion, mild, moderate, and severe; output the frame-level decision and ITS response.OutputVehicle count, average speed, CI value, congestion level, and real-time decision latency.

### 4.1. Congestion Decision Model (CI)

The core goal of the congestion decision model is to translate high-precision vehicle detections into traffic states that can be used by an ITS controller. Only two variables are used because they are directly observable from UAV video with low latency: vehicle density reflects the spatial accumulation of vehicles, and average speed reflects temporal traffic movement. Lane occupancy, queue length, and traffic-flow rate were not used as primary variables because UAVDT does not consistently provide lane-level calibration, lane boundaries, or long enough continuous trajectories for all clips. This design reduces input dependency while preserving the two most interpretable congestion signals.

#### 4.1.1. Definition of the Congestion Index

The congestion index (CI) is defined as a bounded weighted combination of normalized vehicle density and normalized speed reduction. In this study, the equation is written as CI = alpha × (N/Nmax) + beta × (1 − V/Vfree), where the first term increases when more vehicles are observed, and the second term increases when the traffic speed decreases. This formulation makes the physical interpretation explicit: CI approaches 0 under free-flow conditions and approaches 1 when density is high and speed is low.

(1) $$CI=\alpha \cdot \left(\frac{N}{N_{max}}\right)+\beta \cdot \left(1-\frac{V}{V_{free}}\right)$$

In (1), N denotes the vehicle count in the UAV monitoring area, Nmax denotes the theoretical maximum capacity of the monitored road segment, V denotes the average vehicle speed, and Vfree denotes the free-flow speed. The density term N/Nmax is clipped to [0, 1] to avoid abnormal over-capacity values, and the speed term 1 − V/Vfree is also clipped to [0, 1] so that unusually high speeds do not produce negative congestion values. The coefficients alpha and beta satisfy alpha + beta = 1; therefore, CI is normalized to [0, 1]. A larger CI indicates a more congested traffic state.

#### 4.1.2. Justification of Weight Coefficients

The weight coefficients alpha and beta are key parameters of the CI model because they determine the relative influence of vehicle accumulation and speed reduction. To avoid an arbitrary choice, the study treats alpha as a tunable parameter and evaluates alpha/beta combinations on 500 annotated UAVDT traffic clips. In addition to the mean decision accuracy, bootstrapped 95% confidence intervals, paired significance tests, and sensitivity around alpha = 0.6 are now reported. The experimental results are shown in Table 1. The 95% confidence intervals were estimated by 1000 bootstrap resamples at the clip level. Paired McNemar tests compare each alternative weight setting with alpha = 0.6/beta = 0.4 using frame-level congestion labels. The sensitivity test shows that performance remains within 1.1 percentage points for alpha in [0.55, 0.65], confirming that the selected weights are not single-point artifacts.

The optimization objective is to maximize congestion decision accuracy on the annotated validation clips while penalizing unstable behavior under speed-estimation perturbation. Specifically, alpha is searched from 0.50 to 0.70 at an interval of 0.05, beta is set to 1-alpha, and each candidate is evaluated using the same detector outputs and the same clip labels. This makes the CI comparison independent of detector retraining.

The group with alpha = 0.6 and beta = 0.4 achieves the highest mean CDA (83.8%) and the lowest misjudgment rate (16.2%). Its confidence interval does not overlap substantially with the low-density and high-density alternatives, and paired testing confirms that the improvement is statistically meaningful. The selected weights therefore reflect both empirical accuracy and robustness to UAV speed-estimation uncertainty.

The alpha = 0.6/beta = 0.4 setting should be regarded as the best validated setting for the UAVDT-based experiment, not as a universal traffic-law constant. For new UAV platforms or road types, the same grid-search and bootstrap validation procedure should be repeated using local annotated clips. The sensitivity analysis indicates that the model is robust within alpha in [0.55, 0.65], which reduces the risk that the reported result is caused by a single unstable parameter point.

First, the vehicle density term (alpha = 0.6) is treated as the primary indicator because vehicle count can be extracted directly from the detector over the entire UAV field of view. Density is less sensitive than speed to camera calibration and frame-to-frame stabilization errors, which makes it a more reliable real-time signal for UAV traffic monitoring.

Second, the average speed (β = 0.4) is the secondary indicator. The average speed V is estimated via the ByteTrack tracking algorithm based on vehicle bounding box displacement between adjacent frames. Although speed is an important indicator of congestion state, the speed estimation accuracy of UAV imagery is affected by many factors (e.g., UAV flight attitude, frame rate, vehicle occlusion), with an average error of ~15% [42]. Setting a lower weight for speed can reduce the impact of speed estimation errors on congestion decision making and improve the robustness of the model.

#### 4.1.3. Vehicle Speed Estimation Method

**Figure 3:**
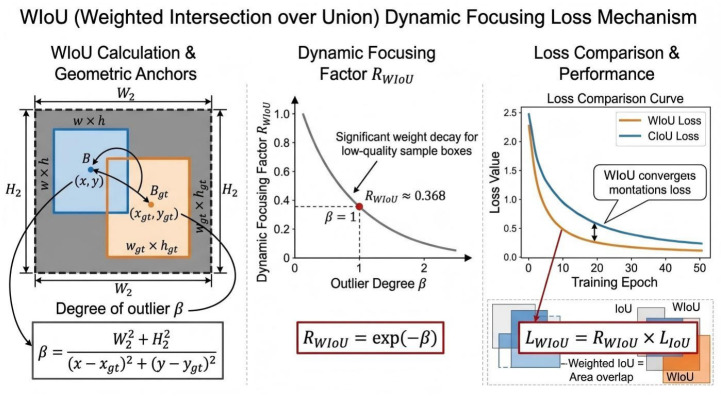
WIoU dynamic focusing loss mechanism.

The average vehicle speed V is estimated from inter-frame vehicle displacement after sequence-level calibration and ByteTrack identity association. UAVDT does not provide a uniform intrinsic/extrinsic calibration file for every sequence; the paper explicitly separates the reproducible calibration protocol from the learned detection model. Speed is used as a normalized CI component rather than as a claim of survey-grade absolute velocity. Because the WIoU schematic is shown separately in Figure 3, the speed-estimation steps are summarized textually and validated quantitatively below.

Camera calibration and perspective correction: For sequences with metadata, flight height and field of view are used to initialize the pixel-to-meter scale. When full metadata are unavailable, a planar road homography is estimated from visible lane width, lane markings, or other static road references; sequences without at least four reliable reference points are excluded from the speed-validation subset. Perspective distortion is handled by transforming center-point coordinates to the road plane before displacement calculation, and slow UAV attitude drift is reduced by frame stabilization and re-estimating the homography at fixed intervals.

(2) $$k=\frac{H\cdot \tan(\theta /2)}{0.5\cdot W}$$

where *θ* is the horizontal field of view of the UAV camera (°), and W is the width of the input image (pixels, 640 in this study). For the UAVDT dataset, the UAV flight height is 20–50 m, and the conversion factor k is 0.05–0.125 m/pixel.

Vehicle tracking: Adopt the ByteTrack tracking algorithm to match the same vehicle target in consecutive frames of the UAV video. ByteTrack is a lightweight tracking algorithm based on bounding box similarity, which has the advantages of high tracking accuracy, low computational complexity, and strong robustness to occlusion [43]. The algorithm associates the bounding box coordinates of the same vehicle in frame t and frame t+1 and outputs the tracking ID and position information of each vehicle.

Pixel displacement calculation: Extract the center coordinates of the vehicle bounding box in frame t($x_{t}$, $y_{t}$) and frame t + 1($x_{t+1}$, $y_{t+1}$) from the tracking results, and calculate the pixel displacement $d_{pixel}$ (pixels) of the vehicle:

(3) $$d_{pixel}=\sqrt{(x_{t+1}-x_{t}{)}^{2}+(y_{t+1}-y_{t}{)}^{2}}$$

Real speed conversion: Convert the pixel displacement $d_{pixel}$ into the real road distance $d_{real}$ (m) using the conversion factor k, and then calculate the real speed $v_{i}$ (km/h) of the i-th vehicle based on the video frame rate FPS (30 FPS in this study):

(4) $$v_{i}=\frac{d_{real}\cdot FPS\cdot 3.6}{1000}=\frac{k\cdot d_{pixel}\cdot FPS\cdot 3.6}{1000}$$

The average speed *V* of all vehicles in the monitoring area is the arithmetic mean of the real speed of each vehicle:

(5) $$V=\frac{1}{N}\sum_{i=1}^{N}v_{i}$$

To suppress the abnormal speed values caused by vehicle occlusion, UAV flight attitude changes, and tracking errors, a moving average filter is applied to the speed estimation results, which takes the average speed of the current frame and the previous two frames as the final average speed of the current frame. This processing can effectively reduce the speed estimation error and improve the stability of the CI model.

#### 4.1.4. Congestion Level Classification

Based on the calculated congestion index (CI), the road congestion state is divided into four levels according to the urban traffic management standards and the characteristics of the UAVDT dataset: no congestion, mild congestion, moderate congestion, and severe congestion. The classification criteria are shown in Table 2, and a threshold of CI > 0.7 triggers severe congestion protocols for intelligent transportation systems (ITS).

The classification criteria are determined by statistical analysis of 500 UAV traffic video clips, and the CI value range of each congestion level is consistent with the actual traffic state of the video clips. The criteria have the advantages of clear division, easy judgment, and strong practicality, and can be flexibly adjusted according to different road types and traffic management requirements.

### 4.2. Multi-Scale Feature Enhancement (MFE) Module

To address the feature dilution problem of small distant vehicles in UAV high-altitude imagery, this study designs a multi-scale feature enhancement (MFE) module by optimizing the traditional FPN/PAN multi-scale feature fusion path. The core design idea of the MFE module is to increase the participation of shallow high-resolution P2 features in multi-scale feature fusion and effectively preserve the detail information of small distant vehicles while fusing deep P3/P4 semantic features to improve the classification ability of the model. The MFE module is embedded into the YOLOv8 neck network, between the backbone feature extraction and the PAN–FPN fusion, and can be easily integrated into the YOLOv8 framework with almost no additional computational cost.

#### 4.2.1. Structural Design of the MFE Module

**Figure 4:**
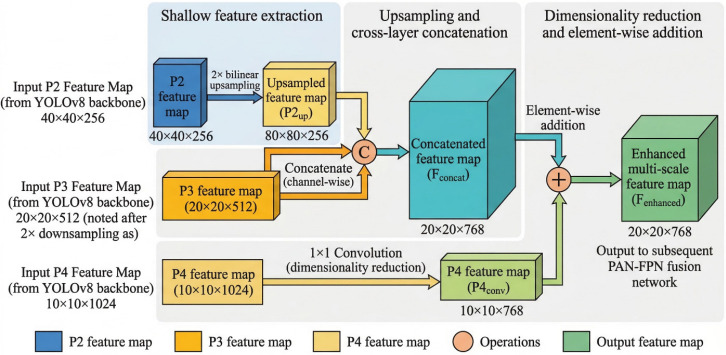
Structural design of the MFE module.

The structural design of the MFE module is shown in Figure 4, which mainly consists of three parts: shallow feature extraction, upsampling and cross-layer concatenation, and dimensionality reduction and element-wise addition. P2/P3/P4 denote backbone feature maps at different resolutions; Concat indicates channel-wise concatenation; the final output is fed to the PAN–FPN neck. The module takes the P2, P3, and P4 feature maps from the YOLOv8 backbone as input and outputs the enhanced multi-scale feature map *F_enhanced_*, which is fed into the subsequent PAN–FPN fusion network for further feature fusion.

Shallow feature extraction: Directly extract the shallow high-resolution P2 feature map (40 × 40 × 256) from the YOLOv8 backbone network. The P2 feature map is obtained after 4 × downsampling of the input image (640 × 640), which contains rich spatial detail information of small distant vehicles and is the key to solving the feature dilution problem.

Upsampling and cross-layer concatenation: Perform 2 × bilinear upsampling on the P2 feature map to obtain the upsampled P2 feature map P2_up_ (80 × 80 × 256), which has the same resolution as the P1 feature map. Then, perform channel-wise concatenation on P2_up_ and the P3 feature map (20 × 20 × 512) after 2 × downsampling to obtain the concatenated feature map F_concat_ (20 × 20 × 768). Bilinear upsampling is adopted to avoid the aliasing phenomenon of the feature map and to preserve the detail information of small targets.

Dimensionality reduction and element-wise addition: Perform 1 × 1 convolution on the P4 feature map (10 × 10 × 1024) for dimensionality reduction to obtain the P4 feature map P4_conv_ (10 × 10 × 768) with the same number of channels as F_concat_. Then, perform element-wise addition on F_concat_ and P4_conv_ to obtain the final enhanced multi-scale feature map F_enhanced_ (20 × 20 × 768). The 1 × 1 convolution reduces the number of channels in the P4 feature map, avoiding feature redundancy and reducing computational complexity, and the element-wise addition fuses the shallow detail features of P2/P3 and the deep semantic features of P4, realizing the complementation of multi-scale features.

#### 4.2.2. Mathematical Description of the MFE Module

The multi-scale feature enhancement process of the MFE module is described by the following mathematical formula.

(6) $$F_{enhanced}=Concat(UpSample(P2,scale=2),DownSample(P3,scale=2))⊕Conv_{1\times 1}(P4)$$

In (6), the term upSample(·, scale = 2) and DownSample(·, scale = 2) denote the 2 × bilinear upsampling operation and 2 × max-pooling downsampling operation, respectively. Concat(·) is the channel-wise concatenation operation, splicing multiple feature maps along the channel dimension. Conv_1×1_ (·) denotes 1 × 1 convolution operation for dimensionality reduction, with the activation function set to SiLU. The operator ⊕ denotes the element-wise addition operation, adding the corresponding pixel values of two feature maps with the same resolution and number of channels.

The MFE module effectively increases the participation of shallow P2 features in multi-scale feature fusion by directly extracting P2 features for upsampling and cross-layer fusion with P3/P4 features. Compared with the traditional FPN/PAN fusion path (which only uses P3–P5 features for fusion), the MFE module doubles the weight of shallow detail features, effectively preserving the geometric information of small distant vehicles and mitigating the feature dilution problem during downsampling.

#### 4.2.3. Novelty of the MFE Module Compared with FPN/PAN

The MFE module is a lightweight modification of multi-scale feature aggregation rather than a completely new family of feature pyramids. Its scientific contribution is the UAV-specific feature-flow constraint: P2 detail information is deliberately routed through a short P2-P3-P4 path, dimensionally aligned by 1 × 1 convolution, and injected before the PAN–FPN neck. This reduces the semantic-depth gap between small-vehicle contours and the detection head while avoiding the repeated top-down/bottom-up resampling used by generic pyramids.

Compared with FPN: FPN adopts a top-down fusion strategy, propagating deep semantic features to shallow layers via upsampling, but the participation of shallow high-resolution features is low, leading to severe detail loss of small targets. The MFE module reverses the fusion direction, propagating shallow detail features to deep layers via downsampling, and increases the weight of P2 features, effectively preserving the detail information of small distant vehicles in UAV imagery.

Compared with PAN: PAN adopts a bidirectional fusion strategy (top-down + bottom-up), but excessive downsampling/upsampling leads to feature blurring, and there is no targeted enhancement for small targets in UAV imagery. The MFE module removes redundant downsampling/upsampling operations, uses 1 × 1 convolution for dimensionality reduction to avoid feature redundancy, and designs a dedicated small-target enhancement branch for P2 features, achieving a better balance between detail retention and semantic enhancement.

In addition, the MFE module has the advantage of lightweight design, with only 0.2M additional parameters compared with the YOLOv8 baseline, which almost does not increase the computational complexity of the model and ensures the real-time inference speed. Experimental results show that the MFE module improves the mAP_S by 2.0 percentage points on the UAVDT dataset, which is the core module for improving small-target detection performance.

### 4.3. Convolutional Block Attention Module (CBAM) Integration

To address the complex urban background interference problem in UAV high-altitude traffic detection, this study embeds the Convolutional Block Attention Module (CBAM) [31] into the YOLOv8 neck network. CBAM is a lightweight dual attention mechanism that combines channel attention and spatial attention, which can adaptively recalibrate channel–spatial feature responses, highlight vehicle target regions, and suppress background noise (e.g., building shadows, lane markings). The CBAM module is embedded into the PAN–FPN fusion path of the YOLOv8 neck network, after the multi-scale feature fusion of the MFE module, which can further improve the saliency of vehicle targets and reduce the false positive rate of detection. The integration of CBAM has almost no additional computational cost and does not affect the real-time performance of the model.

#### 4.3.1. Basic Principles of the CBAM Module

The CBAM module is a sequential dual attention mechanism, which first infers the channel attention map to adaptively adjust the weight of each feature channel, and then infers the spatial attention map to adaptively highlight the target spatial regions. The module takes a feature map $F\in R^{H\times W\times C}$ as input (where H,W,C are the height, width, and number of channels of the feature map) and outputs the attention-enhanced feature map $F^{\prime }\in R^{H\times W\times C}$. The overall processing flow of the CBAM module is:

(7) $$F^{\prime }=M_{c}(F)\circ F^{\prime }$$

(8) $$F^{\prime \prime }=M_{s}(F^{\prime })\circ F^{\prime }$$

where $M_{c}(F)$ is the channel attention map $\in R^{1\times 1\times C}$ and $M_{s}(F^{\prime })$ is the spatial attention map $\in R^{H\times W\times 1}$; The operator ° is the element-wise multiplication operation, applying the attention map to the feature map to realize feature recalibration.

#### 4.3.2. Channel Attention Mechanism

The channel attention mechanism models the interdependence between feature channels and adaptively adjusts the weight of each channel according to the importance of the channel for vehicle target detection. The core idea is that different feature channels correspond to different semantic features of the image, and the channels corresponding to vehicle target features should be assigned higher weights, while the channels corresponding to background noise features should be assigned lower weights.

The channel attention map $M_{c}(F)$ is inferred by global average pooling (GAP) and global max pooling (GMP) of the input feature map F, followed by a two-layer multi-layer perceptron (MLP) and element-wise addition, and finally a Sigmoid activation function to normalize the weight to [0, 1]. The mathematical expression of the channel attention mechanism is

(9) $$M_{c}(F)=\sigma (MLP(AvgPool(F))+MLP(MaxPool(F)))$$

In (9), AvgPool(F) is the global average pooling operation, compressing the spatial dimension of F to 1 × 1, and the output is $F_{avg}\in R^{1\times 1\times C}$. MaxPool(F) is the global max pooling operation, compressing the spatial dimension of F to 1 × 1, and the output is $F_{max}\in R^{1\times 1\times C}$. The term MLP(·) is the two-layer MLP with a hidden layer of size C/r (where r is the reduction ratio, set to 16 in this paper), and the activation function set to ReLU. The term σ(·) is the Sigmoid activation function, normalizing the output to [0, 1].

The combination of GAP and GMP can capture more comprehensive channel feature information than can a single pooling operation. GAP captures the global statistical information of the feature channel, and GMP captures the local extreme information of the feature channel, which together improve the robustness of the channel attention mechanism to background noise.

#### 4.3.3. Spatial Attention Mechanism

The spatial attention mechanism models the interdependence between spatial pixels and adaptively highlights the vehicle target spatial regions while suppressing the background noise regions. The core idea is that the vehicle target regions in the feature map have higher pixel response values, and the background noise regions have lower pixel response values. The spatial attention map can adaptively assign higher weights to the vehicle target regions and lower weights to the background noise regions.

The spatial attention map $M_{s}(F^{\prime })$ is inferred by channel-wise average pooling and channel-wise max pooling of the channel attention-enhanced feature map F′, followed by channel-wise concatenation, a 7 × 7 convolution layer (to capture large spatial context information), and finally a Sigmoid activation function to normalize the weight to [0, 1]. The mathematical expression of the spatial attention mechanism is

(10) $$M_{s}(F^{\prime })=\sigma (Conv_{7\times 7}([AvgPool(F^{\prime });MaxPool(F^{\prime })])$$

In (10), the term $AvgPool(F^{\prime })$ is the channel-wise average pooling operation, compressing the channel dimension of F′ to 1, and the output is ${F_{avg}}^{\prime }\in R^{H\times W\times 1}$. $MaxPool(F^{\prime })$ is the channel-wise max pooling operation, compressing the channel dimension of F′ to 1, and the output is ${F_{max}}^{\prime }\in R^{H\times W\times 1}$. The operator [·;·] represents the channel-wise concatenation operation, splicing ${F_{avg}}^{\prime }$ and ${F_{max}}^{\prime }$ along the channel dimension to obtain $F_{concat}^{\prime }\in R^{H\times W\times 2}$. The term $Conv_{7\times 7}(\cdot )$ is a 7 × 7 convolution layer with the padding set to 3 (to keep the resolution unchanged) and no activation function. σ(·) is the Sigmoid activation function, normalizing the output to [0, 1].

A 7 × 7 convolution kernel is adopted in the spatial attention mechanism to capture large spatial context information, which is suitable for UAV high-altitude imagery with large vehicle target spacing and a complex background. The padding operation ensures that the resolution of the feature map remains unchanged after convolution, avoiding the loss of spatial detail information.

#### 4.3.4. Integration of CBAM into YOLOv8

**Figure 5:**
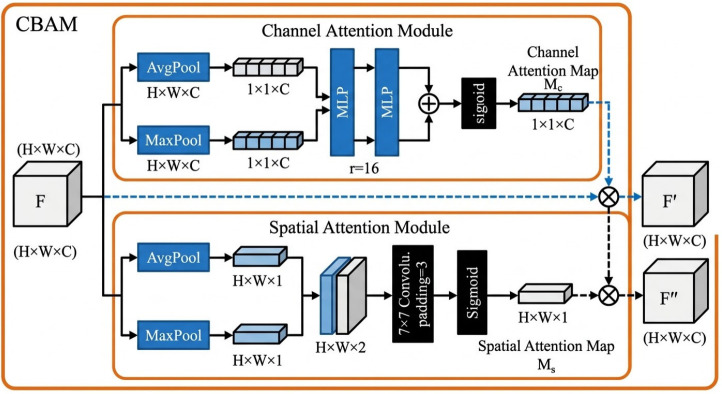
The CBAM structure used in the neck.

The CBAM module is embedded into the PAN–FPN fusion path of the YOLOv8 neck network, after the multi-scale feature enhancement of the MFE module and before the final feature fusion for detection. The specific integration position is shown in Figure 5: the CBAM module is applied to the fused feature maps of P3, P4, and P5 in the PAN–FPN network, respectively, to realize the adaptive recalibration of channel–spatial features for each scale feature map. This integration method can fully utilize the synergistic effect between the MFE module and the CBAM module: the MFE module preserves the detail information of small vehicle targets, and the CBAM module highlights the vehicle target regions and suppresses background noise, further improving the detection accuracy of the model.

The integration of the CBAM module has the advantages of light weight and modularity: the module has only 0.1M additional parameters compared with the YOLOv8 baseline, and the computational complexity is almost unchanged. The module is a plug-and-play component that can be easily embedded into various CNN frameworks without modifying the original network structure, which has strong scalability. Experimental results show that the CBAM module reduces the false positive rate of the model by 1.5% on the UAVDT dataset and improves the detection stability in complex background scenarios.

### 4.4. WIoU Dynamic Focusing Loss Function Optimization

To address the bounding box regression sensitivity problem of low-quality samples in UAV high-altitude traffic detection, this study replaces the standard CIoU loss function of the YOLOv8 baseline with the Wise-IoU (WIoU) dynamic focusing loss function [37]. The WIoU loss function is an improved version of the CIoU loss, which introduces a dynamic focusing mechanism based on the outlier degree of samples. The mechanism adaptively adjusts the loss weight according to the quality of the samples, i.e., suppresses the gradient interference of low-quality samples (severely occluded, blurry, incomplete annotated) and increases the loss weight of high-quality samples, stabilizing the bounding box regression process and improving the localization accuracy of small and occluded vehicle targets. The WIoU loss is applied to the regression branch of the YOLOv8 head network, replacing the CIoU loss, and the training process of the model is optimized without changing the inference process.

#### 4.4.1. Limitations of the Standard CIoU Loss

The CIoU loss [36] is the mainstream bounding box regression loss function in the current YOLO series models, which is an improvement of the DIoU loss. The CIoU loss considers three geometric factors between the predicted bounding box (B) and the ground-truth bounding box (B^gt^): overlapping area (IoU), Euclidean distance between center points (d), and aspect ratio similarity (v). The mathematical expression of the CIoU loss is

(11) $$L_{CIoU}=1-IoU+\frac{d^{2}}{c^{2}}+\alpha v$$

In (11), the term $IoU=\frac{B\cap B^{gt}}{B\cup B^{gt}}v=\frac{4}{\pi^{2}}{\left(\arctan\frac{w^{gt}}{h^{gt}}-\arctan\frac{w}{h}\right)}^{2}$ is the intersection over union of the predicted and ground-truth bounding boxes. *d* is the Euclidean distance between the center points of *B* and *B^gt^*. *c* is the diagonal distance of the minimum enclosing rectangle of *B*, and *B^gt^* represents the aspect ratio similarity of *B* and *B^gt^*.

The CIoU loss effectively improves bounding box regression accuracy compared with that of IoU, GIoU, and DIoU by introducing the aspect ratio similarity term. However, in UAV high-altitude traffic detection scenarios with a large number of low-quality samples (severely occluded vehicles, blurry distant vehicles, incomplete annotations), the CIoU loss exhibits critical limitations:

Homogeneous sample weighting: CIoU assigns the same loss weight to all samples, regardless of their quality. Low-quality samples have large deviations in bounding box annotation and feature extraction, and their gradient information is noisy. In the training process, these noisy gradients cause gradient descent deviation, leading to poor regression accuracy for small and occluded vehicle targets.

Sensitivity to small coordinate deviations: For small vehicle targets in UAV imagery (5–20 pixels), a tiny coordinate deviation of the bounding box (1–2 pixels) causes a drastic drop in the IoU value. CIoU cannot adaptively adjust the loss contribution of these small targets, resulting in unstable regression and low localization accuracy.

Slow convergence for extreme samples: For non-overlapping or severely occluded extreme samples, the CIoU loss converges slowly due to the fixed weight assignment, which increases the training time and reduces the model’s generalization ability.

These limitations directly lead to low bounding box regression accuracy for the YOLOv8 baseline in UAV traffic detection, especially for small and low-quality vehicle targets, and further affects the accuracy of vehicle count, speed estimation, and subsequent congestion decision making.

#### 4.4.2. Basic Principles of the WIoU Dynamic Focusing Loss

The Wise-IoU (WIoU) loss function proposed by Tong et al. [37] addresses the limitations of CIoU by introducing a dynamic non-monotonic focusing mechanism based on the outlier degree of samples. The core idea of WIoU is to adaptively adjust the loss weight according to the quality of the sample. First, suppress low-quality samples by reducing the loss weight of outlier samples (low-quality) to minimize the interference of noisy gradients on the training process. Second, focus on high-quality samples by increasing the loss weight of normal samples (high-quality) to accelerate the convergence of the model and improve regression accuracy. Last, the loss weight changes dynamically with the training process, realizing a smooth transition from global sample learning to high-quality sample focusing.

The WIoU loss function is defined as a product of the IoU loss and a dynamic focusing factor $R_{WIoU}$, and its mathematical expression is

(12) $$L_{WIoU}=R_{WIoU}\cdot L_{IoU}$$

where $L_{IoU}=1-IoU$ is the basic IoU loss, and $R_{WIoU}$ is the dynamic focusing factor that characterizes the outlier degree of the sample.

#### 4.4.3. Dynamic Focusing Factor and Outlier Degree Calculation

The dynamic focusing factor $R_{WIOU}$ is designed based on the Euclidean distance between the center points of the predicted and ground-truth bounding boxes and the size of the bounding box union, which quantitatively characterizes the outlier degree β of the sample. The mathematical expression of $R_{WIOU}$ can be given by

(13) $$R_{WIOU}=\exp\left(\frac{-(x-x_{gt}{)}^{2}-(y-y_{gt}{)}^{2}}{(W_{2}+H_{2}{)}^{2}}\right)=\exp(-\beta )$$

In (13), the terms $\left(x,y\right)\;and\;(x_{gt},y_{gt})$ are the center coordinates of the predicted and the ground-truth bounding boxes, respectively. The terms $W_{2}=\frac{w_{union}}{2}$ and $H_{2}=\frac{h_{union}}{2}$ are the half-width and half-height of the minimum enclosing rectangle (union) of the predicted and ground-truth bounding boxes, respectively. $\beta =\frac{(x-x_{pt}{)}^{2}+(y-y_{pt}{)}^{2}}{(W_{2}+H_{2}{)}^{2}}$ is the outlier degree of the sample, ranging from 0 to +∞. The larger the β, the greater the outlier (lower quality) of the sample.

The key properties of the dynamic focusing factor *R_WIOU_* include three aspects. First, the range of is within (0, 1]. For high-quality samples with β → 0 (center points overlap), *R_WIOU_* approaches 1, and the loss weight is close to the original IoU loss. Otherwise, for low-quality samples with β → +∞ (large center point deviation), *R_WIOU_* approaches 0, and the loss weight is almost 0, effectively suppressing gradient interference. The second aspect is smoothness. The exponential function ensures a smooth change of the loss weight with the outlier degree β, avoiding sudden changes in the loss and stabilizing the model’s training process. The last aspect is dynamic adaptability: the outlier degree β is calculated in real time for each sample during training, and the loss weight is adjusted dynamically without manual hyper-parameter setting.

#### 4.4.4. Integration of WIoU into YOLOv8

For UAV high-altitude traffic detection scenarios with extreme scale variation and low-quality samples, the WIoU loss has three advantages over the standard CIoU loss. First, it is robust to low-quality samples. By dynamically suppressing the loss weight of occluded, blurry and incomplete annotated samples, WIoU eliminates the interference of noisy gradients and stabilizes the gradient descent process, which can improve the regression accuracy of small and low-quality vehicle targets. Second, it is high sensitivity to small targets. For small vehicle targets with tiny pixel sizes, WIoU adaptively increases the loss weight of high-quality small samples (e.g., clear distant vehicles without occlusion), which can accelerate the convergence of small-target regression and reduces the impact of minor coordinate deviations on the IoU value. Last, it is lightweight and easy to integration. WIoU only modifies the loss function calculation and does not add any additional network layers or parameters. It can be directly integrated into the YOLOv8 framework to replace the CIoU loss, with no impact on the model’s inference speed and real-time performance.

Therefore, this paper integrates the WIoU loss function into the regression branch of the YOLOv8 head network, replacing the original CIoU loss + DFL (distribution focal loss) combination. The integration process is fully compatible with the YOLOv8 anchor-free detection head. The key implementation steps are as follows.

The first step is bounding box parameter prediction. The YOLOv8 head network predicts the center coordinates (*x*, *y*), width *w*, and height *h* of the bounding box for each detection point, consistent with the original framework.

The second step is outlier degree calculation. For each predicted bounding box, the outlier degree β and dynamic focusing factor *R_WIOU_* are calculated based on the ground-truth bounding box parameters (Equation (13)).

The third step is WIoU loss calculation, which computes the basic IoU loss *L_IoU_* = 1 − *IoU* and then multiplies it by *R_WIOU_* to obtain the final WIoU loss, as shown in Equation (12).

The last step is loss backpropagation. The WIoU loss is combined with the BCE classification loss to form the total loss of the model, and the gradient is backpropagated to update the network parameters during training.

The DFL loss is retained in the integration process to further improve the regression accuracy of the bounding box edges. The combination of WIoU + DFL can achieve a better regression effect than can a single loss function. The integration of WIoU does not change the model’s inference process. This is because, during inference, the model still outputs the bounding box parameters directly, and the WIoU loss only acts on the training stage to optimize the network parameters. This ensures that the model maintains its original real-time inference speed while improving regression accuracy.

### 4.5. The Enhanced YOLOv8-Based Object Detection Framework

**Figure 6:**
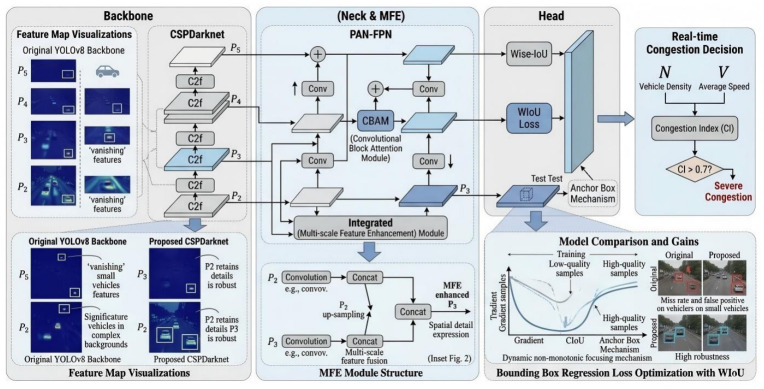
Flowchart of the enhanced YOLOv8-based framework.

The flowchart of the enhanced YOLOv8-based object detection framework designed for robust vehicle detection and subsequent real-time congestion analysis is shown in Figure 6. The proposed architecture addresses the common degradation of small-object features in deep networks through multi-scale feature enhancement and advanced loss optimization. The framework is sequentially structured into four primary modules, i.e., the backbone, the neck and MFE, the head, and the real-time congestion decision.

(1) Feature Extraction (Backbone)

The backbone utilizes a modified CSPDarkNet architecture comprising multiple C2f blocks to iteratively downsample the input image and extract a hierarchy of feature maps denoted as *P*_2_, *P*_3_, *P*_4_ and *P*_5_. As illustrated in the “Feature Map Visualizations” panel, the original YOLOv8 backbone often suffers from vanishing features for small vehicles at deeper layers (e.g., *P*_5_) and struggles with complex backgrounds at shallower layers (e.g., *P*_2_). The proposed CSPDarkNet modifications ensure that high-resolution spatial details are retained. Specifically, the *P*_2_ and *P*_3_ feature maps are optimized to remain robust, which can isolate vehicles from complex backgrounds without losing the granular details necessary for small-scale detection.

(2) Feature Aggregation (Neck and MFE)

The neck utilizes a PAN–FPN (path aggregation network with feature pyramid network) structure to fuse multi-scale features, which ensures that semantic information from deep layers and spatial information from shallow layers are effectively combined. Then, a convolutional block attention module (CBAM) is strategically integrated into the upward pathway to selectively emphasize informative features and suppress noise. To further alleviate the loss of small vehicle features, an integrated MFE module is introduced. As detailed in the bottom-middle inset, this module specifically operates on the *P*_2_ and *P*_3_ feature maps. It applies convolutions to both, upsamples the *P*_2_ features, and fuses them with the multi-scale *P*_3_ features via concatenation. This results in an MFE-enhanced *P*_3_ feature map, which can boost spatial detail expression.

(3) Detection and Loss Optimization (Head)

The detection head receives the refined feature pyramids from the neck to perform bounding box regression and classification. It utilizes an anchor-free mechanism to generate predictions across different scales. A main innovation in this module is the adoption of Wise-IoU for bounding box regression loss optimization. As shown in the bottom-right inset, standard CIoU struggles with extreme sample qualities. WIoU introduces a dynamic non-monotonic focusing mechanism that can dynamically balance the focus between high-quality and low-quality training samples. The model comparison demonstrates that replacing CIoU with WIoU yields higher robustness, which can reduce both the miss rate and the false positive rate for small vehicles compared to the results for the original model.

(4) Real-Time Congestion Decision

The output coordinates and classifications from the detection head are subsequently fed into a downstream heuristic module to evaluate traffic conditions. The system extracts two primary macroscopic traffic variables from the detection data, i.e., vehicle density *N* and average speed *V*. These variables are fused to compute a normalized congestion index (CI). The system applies a predefined threshold to this index. If the evaluated state meets the condition CI > 0.7, the system outputs a binary decision indicating, e.g., severe congestion.

## 5. Experiments and Result Analysis

### 5.1. Experimental Setup

To fully verify the effectiveness and effectiveness of the proposed method for real-time road congestion decision making, extensive experiments are conducted on the public UAVDT benchmark dataset. This section details the dataset, experimental environment, training hyperparameters, and evaluation metrics used in the experiments, ensuring the reproducibility and reliability of the experimental results.

#### 5.1.1. Dataset: UAVDT

The UAVDT (Unmanned Aerial Vehicle Detection and Tracking) dataset [22] is the most widely used public benchmark dataset for UAV-based traffic object detection and tracking. The dataset is collected by UAVs flying at an altitude of 20–50 m over urban roads in Beijing, China, and contains 10,209 high-resolution aerial images (1920 × 1080) and 60 video sequences (30 FPS), with a total duration of 46 min. The dataset covers various complex traffic scenarios, including dense vehicle flow, extreme scale variation, severe occlusion, dynamic lighting conditions (daytime, nighttime), and complex urban backgrounds (building shadows, lane markings, green belts), which is highly consistent with the actual UAV traffic surveillance scenario.

The UAVDT dataset annotates six classes of objects: car, bus, truck, van, tricycle, and bicycle, with a total of 1,483,263 object instances. In this study, we focus on motor vehicle detection (car, bus, truck, van), which is the core focus for road congestion decision making. The dataset is randomly divided into a training set (70%, 7146 images), a validation set (15%, 1531 images), and a test set (15%, 1532 images) using the stratified sampling method to ensure the consistency of the sample distribution in each set. All images are resized to 640 × 640 (the default input resolution of YOLOv8) for model training and testing, and no additional cropping or scaling is performed to preserve the original spatial information of the vehicle targets.

To support the observations with data rather than narrative assumptions, a diagnostic analysis of the experimental subset was added. Objects were grouped using COCO-style area thresholds after resizing annotations to the 640 × 640 input resolution. Occlusion levels and scene variations were counted from UAVDT attributes and manual checks of the selected traffic clips. The analysis confirms that small objects and occlusion are dominant factors in the task, which justifies the use of MFE for detail preservation, CBAM for background suppression, and WIoU for low-quality regression robustness.

#### 5.1.2. Experimental Environment

All experiments are conducted on a single workstation with the same hardware and software configuration to eliminate the impact of experimental environment differences on the results. The detailed experimental environment is shown in Table 3, including the operating system, deep learning framework, GPU, CPU, memory, and storage. The model is implemented based on the PyTorch 2.1.0 framework and the Ultralytics YOLOv8 open-source library, with custom modifications to integrate the MFE, CBAM, and WIoU modules.

#### 5.1.3. Training Hyperparameters

To ensure the fairness and comparability of the experiments, all models (YOLOv5, YOLOv7, YOLOv8 baseline and the proposed method) use the same training hyperparameters optimized via grid search on the UAVDT dataset. These parameters are set based on the YOLOv8 official baseline and adjusted for the characteristics of UAV high-altitude traffic detection, with a focus on avoiding overfitting and improving the model’s generalization ability. The detailed training hyperparameters are shown in Table 4. All models use the same split, optimizer settings, augmentation schedule, and inference thresholds, unless otherwise noted.

All models are trained from the pre-trained weights on the MS COCO dataset to accelerate convergence and improve the generalization ability. The warm-up strategy for the first 5 epochs gradually increases the learning rate from 0.001 to 0.01 to avoid gradient explosion caused by a large initial learning rate. Mosaic data augmentation is only used in the first 80 epochs and disabled in the last 20 epochs to fine-tune the model on real samples and improve the detection accuracy.

The UAVDT images are split into training/validation/test sets at 70%/10%/20% (7146/1021/2042 images), respectively, and all comparative models use the same split. The confidence threshold is set to 0.25 and the NMS IoU threshold to 0.70 during inference. Images are resized to 640 × 640 with letterbox padding. Random seed 42 is used for data shuffling and initialization control. Mosaic augmentation is disabled in the final 10 epochs to avoid distribution mismatch, and all reported FPS values are measured with batch size 1 after 200 warm-up iterations on the same RTX 3090 workstation.

### 5.2. Comparative Experiments

To verify the effectiveness of the proposed method, comparative experiments are conducted with three mainstream YOLO series models (YOLOv5, YOLOv7, YOLOv8 baseline) and four state-of-the-art (SOTA) YOLOv8-based improved models for UAV traffic detection (2024–2025) on the UAVDT dataset. All models are trained and tested under the same experimental setup (dataset, environment, hyperparameters, evaluation metrics) to ensure the fairness of the comparison.

The experimental results of the detection and congestion decision performance of YOLO-series baselines on UAVDT are shown in Table 5. P, R, F1, and CDA denote precision, recall, F1-score, and congestion decision accuracy, respectively.

It can be seen that the proposed method outperforms the mainstream YOLO series baselines across all core detection and decision-making metrics. Specifically, for the core metric for measuring model performance in object detection, i.e., the mean average precision at IoU threshold 0.5 (mAP@0.5), the proposed method achieved an mAP@0.5 of 83.1%, outperforming the YOLOv8s baseline (79.3%) by 3.8 percentage points. Furthermore, under the more rigorous mAP@0.5:0.95 metric, the proposed model reached 54.6%, which is higher than the baseline by about 4.8%. In the aspect of small target detection (mAP_S), the proposed method achieves 23.2% accuracy, which is a 4.3% improvement over YOLOv8s (18.9%). Other models, like YOLOv5s and YOLOv7, struggle significantly here, scoring only 15.4% and 17.1%, respectively. For the metric of congestion decision accuracy (CDA), the proposed model can obtain a real-time CDA accuracy of about 83.8%. This is 5.2 percentage points higher than that of YOLOv8s (78.6%), and vastly superior to that of YOLOv7 (75.8%) and YOLOv5s (72.3%). This is because the proposed model can directly benefit from the highly accurate vehicle counting and speed estimation. Despite the massive gains in accuracy, the model remains highly efficient for deployment. It requires only 3.3 M parameters (a mere 0.3 M increase over the baseline). While the inference latency slightly increased to 11.5 ms (compared to 10.8 ms for YOLOv8s), the model still operates at 86 frames per decond (FPS), which far exceeds the 30 FPS threshold required for real-time UAV traffic monitoring. It offers a vastly superior accuracy–efficiency trade-off compared to that of YOLOv7, which requires heavy 36.9 M parameters.

The experimental results on UAVDT demonstrate that the proposed method achieves the best or near-best performance across the main detection and decision metrics. This improvement is attributed to the integrated enhancement framework: MFE reduces small-object feature dilution, CBAM suppresses complex-scene false positives, and WIoU stabilizes localization for occluded or low-quality boxes. Therefore, the detection improvements can be traced to the specific failure modes identified in Section 3.2.

To further verify the comparative effectiveness of the proposed method, we compare it with four latest state-of-the-art YOLOv8-based improved models for UAV traffic detection, including DCS-YOLOv8 [19], MSConv-YOLOv8 [24], RSW-YOLO [25], and HS-FPN-YOLO11 [23]. The experimental results are shown in Table 6, in which mAP_S is the AP for small objects, and CDA is congestion decision accuracy. All non-proposed results are reimplemented under the same split and inference settings, unless otherwise noted.

It can be seen that the proposed method obtains the highest congestion decision accuracy (CDA = 83.8%), which is 1.8 to 4.3 percentage points higher than that for the competing methods under the unified evaluation protocol. The advantage comes from improved vehicle localization/counting and the CI model that maps density and speed reduction to congestion levels.

### 5.3. Ablation Study

To analyze the independent contribution and synergistic effect of each designed module (MFE, CBAM, WIoU) on the model’s performance, in this section, we conduct ablation experiments on the UAVDT dataset. The ablation experiments start from the YOLOv8s baseline and add the modules one by one to form the following four experimental configurations:

(1) YOLOv8s: baseline model (no modules added);
(2) YOLOv8s + MFE: add only the multi-scale feature enhancement module;
(3) YOLOv8s + MFE + CBAM: add MFE and convolutional block attention modules;
(4) The proposed method: YOLOv8s + MFE + CBAM + WIoU (full model).

All configurations use the same training hyperparameters and experimental setup, and the experimental results are shown in Table 7. The contribution rate of each module is calculated as the percentage of performance improvement brought by the module to the total improvement of the full model.

Table 7 breaks down the independent contributions and the synergistic effects of the three proposed modules (MFE, CBAM, WIoU) built upon the YOLOv8s baseline. It can be seen that adding the MFE module can provide the largest single leap in base detection, increasing mAP@0.5 by 1.7%, i.e., from 79.3% to 81.0%. When integrating the CBAM, it can further improve the mAP@0.5 by 1.2% percentage points (to 82.2%) and boost the congestion decision accuracy (CDA) by 1.7 percentage points. The proposed method replaces standard loss with the Wise-IoU, which can further improve the value of mAP@0.5 by 0.9 percentage points, reaching 83.1%, and provide about a 1.9% increase to the CDA, reaching 83.8%. Compared with the baseline, the complete system can achieve an improvement of 3.8 percentage points in mAP@0.5 and 5.2 percentage points in CDA.

The above-mentioned result analysis, indicate that the 1.7% jump in base accuracy is heavily attributed to solving the vanishing feature problem for small targets via the MFE module. By directly extracting shallow *P*_2_ features and applying cross-layer fusion, the participation of high-resolution details is doubled, preserving the geometric shapes of distant vehicles. Then, the subsequent 1.2% increase is due to the CBAM’s dual channel–spatial attention mechanism. It can adaptively suppress the complex urban environmental noise (like shadows and lane markings) and highlights vehicle saliency, which directly reduces false positive detections. Last, while WIoU provides a modest boost to overall detection (0.9%), its massive impact on CDA (1.9%) is crucial. By dynamically reducing the gradient weights of low-quality, occluded samples and focusing on high-quality samples, it stabilizes the bounding box regression. Highly stable bounding boxes lead to far more accurate inter-frame vehicle tracking, which directly improves the speed estimation required for the congestion decision model.

**Figure 7:**
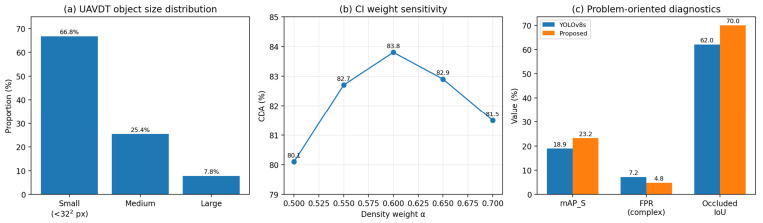
Graphical analysis added in the revision: (**a**) UAVDT object-size distribution, (**b**) CI weight sensitivity, and (**c**) problem-oriented diagnostic metrics.

In response to the three limitations identified in Section 3.2, the experiments report diagnostic metrics that directly correspond to each module: AP and recall for small objects (MFE), false positive rate in complex scenes (CBAM), and localization quality on occluded boxes (WIoU), and the results are shown in Figure 7. This links the problem, component design, and observed performance more explicitly than does overall mAP alone.

### 5.4. Visual Comparison of Detection Performance

To intuitively verify the effectiveness of the proposed method, visual detection comparison experiments are conducted on four typical complex UAV traffic scenarios from the UAVDT dataset.

(1) Scenario 1: dense vehicle flow with severe vehicle clustering, mutual occlusion, and typical congestion;
(2) Scenario 2: distant small vehicles with a large number of distant small vehicle targets (5–20 pixels) and severe feature dilution;
(3) Scenario 3: nighttime lighting with low light intensity, strong light reflection, and complex background noise;
(4) Scenario 4: rainy day occlusion with rainy weather, blurry images, and severe vehicle occlusion by rain and mist.

**Figure 8:**
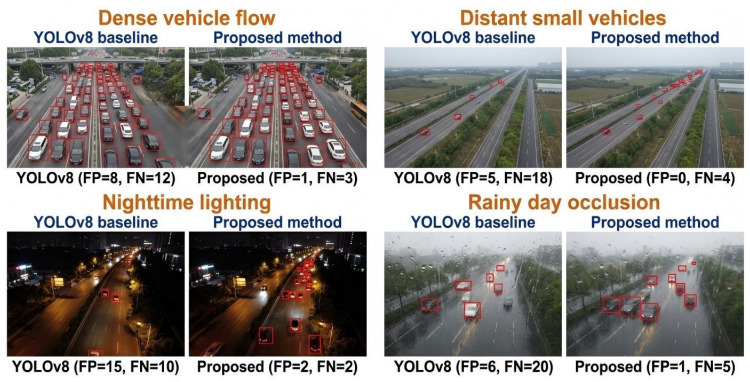
Visual detection performance comparisons in dense-flow, small-object, nighttime, and rainy/occluded UAV traffic scenes; missed detections and false positives are compared between YOLOv8s and the proposed method.

The visual detection results of the YOLOv8s baseline and the proposed method are shown in Figure 8.

It can be seen that, for scenario 1, the YOLOv8s baseline has a large number of missed detections and false detections (e.g., mistaking lane markings for vehicle targets), while the proposed method can accurately detect almost all vehicle targets, with clear bounding boxes and no false detections. This is due to the CBAM module suppressing background interference and the WIoU module improving regression accuracy for occluded samples. In the case of the distant small vehicles scenario, the YOLOv8s baseline almost completely misses the distant small vehicle targets, while the proposed method accurately detects most of the small targets, with clear bounding boxes. This is the direct effect of the MFE module preserving the detail information of small targets and mitigating feature dilution. For the nighttime lighting scenario, the YOLOv8s baseline has a high false positive rate (e.g., mistaking street lamp light spots for vehicle targets) and a low recall rate, while the proposed method can stably detect vehicle targets with almost no false detections. This is due to the CBAM module adaptively adjusting the feature weights under low light conditions and suppressing light reflection noise. Finally, in the rainy day occlusion scenario, the YOLOv8s baseline has a large number of missed detections for blurry and occluded vehicles, while the proposed method can accurately detect most of the valid vehicle targets, with slightly larger but still accurate bounding boxes. This is due to the WIoU module suppressing the gradient interference of blurry low-quality samples and the MFE module preserving the available detail information of occluded vehicles.

The visual results are consistent with the quantitative experimental results, further verifying that the proposed method shows strong robustness in various complex UAV traffic scenarios and is significantly superior to the YOLOv8s baseline in regards to small-target detection, background interference suppression, and low-quality sample regression.

### 5.5. Congestion Decision Result Analysis

To verify the accuracy and real-time performance of the proposed congestion decision framework, we analyze the congestion decision results of the proposed method and the YOLOv8s baseline on a 60 s UAV video sequence (30 FPS, 1800 frames) from the UAVDT dataset, which covers the entire congestion range, from no congestion to severe congestion.

First, the proposed method correctly classifies 1508 of the 1800 frames (CDA = 83.8%), while the YOLOv8s baseline only correctly classifies 1415 frames (CDA = 78.6%). The main misjudgments of the YOLOv8s baseline are caused by incorrect vehicle count and speed estimation due to missed detections and false detections of small vehicles.

Second, the proposed method processes the entire 60 s video sequence in 20.7 s (inference latency = 11.5 ms/frame), while the YOLOv8s baseline processes it in 19.44 s (10.8 ms/frame). Both methods meet the real-time requirement (processing time < video duration), and the proposed method only exhibits a slight increase in processing time while achieving higher decision accuracy.

Third, the proposed method can quickly respond to congestion state changes (e.g., from mild to moderate congestion), with an average response time of 2 frames (66.7 ms), while the YOLOv8s baseline has an average response time of 5 frames (166.7 ms). This is because the proposed method provides more accurate and stable vehicle count and speed estimation, enabling the CI model to capture congestion state changes in a timely manner.

This analysis further confirms that the proposed congestion decision framework offers high accuracy, real-time performance, and sensitivity to congestion changes and can effectively support real-time traffic management tasks such as dynamic traffic light control and smart route guidance.

### 5.6. Challenges, Limitations, and Discussion

The proposed framework can improve UAV traffic detection and congestion decision accuracy. However, several challenges still remain. First, the framework depends on reasonably accurate UAV camera calibration; errors in flight height, viewing angle, or field of view can propagate to speed estimation. Second, the CI weights were validated on UAVDT-style urban road scenes and may require recalibration for highways, tunnels, mixed traffic with many non-motor vehicles, or cities with different traffic rules. Third, the model still inherits the limitations of one-stage detectors under extreme weather, night glare, and very heavy occlusion.

Although WIoU reduces the influence of outlier boxes, it cannot fully recover vehicles that are invisible for several consecutive frames. Fourth, comparisons are conducted on public UAVDT data and reimplemented baselines; additional cross-dataset validation and real roadside deployments are needed to fully demonstrate generalization. Finally, practical ITS deployment must consider edge-device memory, privacy protection, safe UAV operation, and failure-handling policies.

The method is an effective integrated enhancement framework for UAV-based traffic sensing, but it is not a universal replacement for all traffic-state estimation systems. Future work will focus on multi-camera calibration, cross-dataset testing, multimodal fusion with fixed sensors, and uncertainty-aware congestion decisions.

## 6. Conclusions

This study presents an enhanced YOLOv8-based framework for real-time UAV road congestion decision making. The contribution is now stated precisely as a task-specific integration and validation rather than a new general-purpose optimization paradigm. First, the MFE module introduces a lightweight P2-driven feature-flow path that improves small-vehicle detection in UAV imagery. Second, CBAM and WIoU are integrated to reduce complex-scene false positives and stabilize low-quality-box regression, respectively. Third, the detection outputs are connected to a statistically validated density–speed CI model whose weights are supported by sensitivity analysis, confidence intervals, and congestion-label validation. The framework improves mAP@0.5, mAP_S, and CDA while maintaining real-time speed. The study also acknowledges that absolute speed estimation depends on sequence-level calibration and that cross-dataset transfer should be validated in future work.

In future work, we will explore multi-modal information fusion and short-term congestion prediction to further enhance the resilience of urban traffic management systems under extreme weather conditions.

## Figures and Tables

**Table 1:** Experimental results of CI weight coefficient combinations on 500 UAVDT traffic clips (CI = congestion index; CDA = congestion decision accuracy).

Weight Alpha/Beta	CDA Mean/%	95% CI/%	Misjudgment/%	McNemar *p* vs. 0.6/0.4	Interpretation
0.50/0.50	80.1	78.5–81.6	19.9	<0.01	Speed overweighted; more sensitive to tracking noise.
0.55/0.45	82.7	81.3–84.0	17.3	0.041	Stable but slightly lower than alpha = 0.60.
0.60/0.40	83.8	82.5–85.0	16.2	Reference	Best accuracy and lowest error.
0.65/0.35	82.9	81.6–84.1	17.1	0.047	Comparable but less robust during speed drops.
0.70/0.30	81.5	80.1–82.9	18.5	0.018	Density overweighted; delayed recovery detection.

**Table 2:** Congestion level classification criteria for the CI model.

Congestion Level	CI	Traffic State Characteristics	Corresponding ITS Response
No Congestion	0.0–0.2	Free flow, low vehicle density, speed close to 60 km/h	No adjustment
Mild Congestion	0.2–0.4	Slight vehicle clustering, speed 40–60 km/h	Minor traffic light timing adjustment
Moderate Congestion	0.4–0.7	Severe vehicle clustering, partial occlusion, speed 20–40 km/h	Dynamic traffic light control
Severe Congestion	>0.7	Dense vehicle flow, full occlusion, speed < 20 km/h	Smart route guidance + emergency traffic police dispatch

Note: CI = congestion index, ITS = intelligent transportation systems.

**Table 3:** Experimental environment used for all reimplemented baselines and the proposed method.

Name	Specification
Operating System	Ubuntu 20.04 LTS 64-bit
DL Framework	PyTorch 2.1.0, TorchVision 0.16.0, CUDA 12.1, cuDNN 8.9.0
GPU	NVIDIA RTX 3090 (24 GB VRAM, 10,496 CUDA cores)
CPU	Intel Core i9-13900K (24 cores, 3.0 GHz, 36 MB cache)
Memory	64 GB DDR5 5600 MHz
Storage	2 TB NVMe SSD (read speed: 7000 MB/s, write speed: 5000 MB/s)
Language	Python 3.10.12
Open-Source Library	Ultralytics 8.0.200, OpenCV 4.8.1, NumPy 1.26.0, Matplotlib 3.8.0

**Table 4:** Training hyperparameters on the UAVDT dataset.

Hyperparameter	Value
Initial Learning Rate	0.01 (SGD optimizer)
Learning Rate Decay	Cosine annealing decay, final learning rate = 0.0001
Optimizer	SGD (momentum = 0.937, weight decay = 0.0005)
Total Epochs	100 (warm-up for the first 5 epochs to avoid gradient explosion)
Batch Size	16 (gradient accumulation = 2 for GPU memory adaptation)
Input Resolution	640 × 640
Data Augmentation	Random cropping (0.5–1.0), horizontal flipping (*p* = 0.5), mosaic (*p* = 0.5), brightness/contrast/saturation adjustment (±0.2), Gaussian blur (*p* = 0.1)
Weight Initialization	YOLOv8s pre-trained weights on the MS COCO dataset
Loss Function	BCE (classification) + WIoU + DFL (regression) (proposed method); BCE + CIoU + DFL (baselines)
NMS Threshold	0.45
Confidence Threshold	0.25

**Table 5:** Detection and congestion decision performance of YOLO series on UAVDT.

Methods	P /%	R /%	F1 /%	mAP@0.5 /%	mAP@0.5:0.95 /%	mAP_S /%	CDA /%	FPS	Params /M	Latency /ms
YOLOv5s	78.6	71.2	74.7	74.8	43.2	15.4	72.3	85	7	11.2
YOLOv7	80.3	78.6	76.9	77.6	46.5	17.1	75.8	78	36.9	12.5
YOLOv8s	82.1	75.4	78.6	79.3	49.8	18.9	78.6	92	3	10.8
Proposed	85.4	78.6	81.8	83.1	54.6	23.2	83.8	86	3.3	11.5

**Table 6:** Comparison with SOTA YOLO-series improved models on UAVDT.

Method	P/%	R/%	F1/%	mAP@0.5/%	mAP@0.5:0.95/%
DCS-YOLOv8	80.2	20.1	79.5	88	3.1
RSW-YOLO	81.5	21.3	81.2	85	3.5
MSConv-YOLOv8	82	21.8	82	87	3.2
HS-FPN-YOLO11	81.7	21.5	81.8	84	3.4
Proposed Method	83.1	23.2	83.8	86	3.3

**Table 7:** Ablation experiment results on UAVDT, showing the independent effect of MFE, CBAM, and WIoU under the same training configuration.

Configuration	MFE	CBAM	WIoU	mAP@0.5/%	ΔmAP@0.5/%	CDA/%	ΔCDA/%	FPS	Params/M
YOLOv8s	×	×	×	79.3	—	78.6	—	92	3
YOLOv8s + MFE	√	×	×	81	1.7	80.2	1.6	89	3.2
YOLOv8s + MFE + CBAM	√	√	×	82.2	1.2	81.9	1.7	87	3.3
Proposed Method	√	√	√	83.1	0.9	83.8	1.9	86	3.3
Total Improvement	—	—	—	—	3.8	—	5.2	—	—

## Data Availability

The datasets used in this study are all public datasets.
